# COVID‐19 pandemic: Burdens on and consequences for nursing home staff

**DOI:** 10.1111/jan.15193

**Published:** 2022-03-14

**Authors:** Manuela Hoedl, Nina Thonhofer, Daniela Schoberer

**Affiliations:** ^1^ Institute of Nursing Science Medical University of Graz Graz Austria

**Keywords:** COVID‐19, nursing home care, nursing staff

## Abstract

The objective of this study was to assess burdens placed on and consequences of the COVID‐19 pandemic on nursing home staff.

**Design:**

We conducted a qualitative descriptive interview study.

**Methods:**

We interviewed 18 nurses, nursing aides and care aides from five different nursing homes by using a semi‐structured interview guideline between June and September 2020. Data were analysed with a qualitative content analysis method by combining an inductive and deductive coding frame.

**Results:**

Results show that the qualitative work load and work organization were major concerns. Regarding the qualitative work load, participants stated that they were required to perform additional tasks to care for residents, because the pandemic interventions placed the residents under stress and dealing with relatives presented significant challenges. Nursing home staff reported that psychological consequences such as uncertainty, fear and stress represented major effects of the COVID‐19 situation.

**Conclusion:**

We could show that qualitative workloads were assessed and perceived differently. Most nursing home care staff members experienced the changes in working conditions as both physically and psychologically challenging.

**Impact:**

We highly recommend that nursing home staff receive support in such pandemics by being allowed, for example personal protective equipment breaks. Individually tailored programs need to be established to enhance wellbeing and decrease psychological stress and fear in such challenging times.

## 1 INTRODUCTION

Nursing staff, including nurses, nursing aides and care aides play a major role in the nursing home setting (White et al., 2020). As an example, in the nursing home, nurses are responsible for supervising nursing aides, coordinating care, interacting with medical health personnel and planning and providing high‐quality nursing care (Montayre & Montayre, 2017). As nursing homes were a strongly affected setting in the COVID‐19 pandemic (McGilton et al., 2020), nursing staff were key personnel in the fight against the coronavirus and its impacts. Nursing home staff was still required to balance between containing the coronavirus, on the one hand, and maintaining the quality of life of the residents, on the other hand. Numerous measures that were put in place to prevent the spread of infection limited the residents' quality of life and had considerable psychosocial consequences for residents and staff (Benzinger et al., 2021; Strang et al., 2020).

In addition, a group of international scientists highlighted the fact that nursing homes were seldom mentioned when talking about the COVID‐19 pandemic, although a significant proportion of deaths were/are attributed to nursing home residents (McGilton et al., 2020). They also noted that the chronic understaffing in nursing home, heavy workloads, punitive measures related to sick leave, low wages and many other factors were major concerns that were uncovered due to this pandemic (McGilton et al., 2020). Another group of authors highlighted the failure to include nursing homes in a timely manner in the systematic planning of a response to COVID‐19 (O'Neill et al., 2020). To meet this demand, additional studies on the situation in nursing homes are needed. International organizations have highlighted the need to pay more attention to nursing homes during this pandemic to improve care and education, as well as to conduct more relevant research there.

## 2 BACKGROUND

In recent months, several papers have been published on the COVID‐19 pandemic. Most of these papers place a focus on the care of hospital patients. We identified six articles addressing the nursing home setting, and only two of these articles dealt with the situation of staff providing direct nursing home care (Kabir et al., 2020; Sarabia‐Cobo et al., 2021).

The study by Kabir and colleagues represents a brief report on the experiences of one Swedish nurse who was working on the frontline in a Swedish nursing home (Kabir et al., 2020). Sarabia‐Cobo and colleagues conducted a qualitative interview study using a phenomenological approach, exploring the experiences and expectations of nurses as they performed their care duties (Sarabia‐Cobo et al., 2021). Even though this was an international study with participants from four countries, this study only included registered nurses working at a nursing home with positive cases of COVID‐19 among the residents and/or staff (Sarabia‐Cobo et al., 2021). So these studies did not place a focus on the situation of nursing aides or care aides, who comprise the largest proportion of workforce in nursing homes (Harris‐Kojetin et al., 2013). In the context of this study, nursing aides are responsible for, for example repositioning, body hygiene, eating and drinking of care‐dependent residents, whereas, care aides, support residents, that are not completely care dependent, with body hygiene or eating and drinking. Moreover, Sarabia‐Cobo and colleagues also stated that one limitation of their study was the fact, that they had only limited time to analyse the data (Sarabia‐Cobo et al., 2021).

In addition, nursing/care aides, who are often responsible for many tasks, such as the personal hygiene of the residents, were not included, even though all nursing home staff, regardless of their qualifications, had to follow the international protocols and restrictions. Therefore, a holistic picture of the nursing home situation during the COVID‐19 pandemic including the different views of nurses, nursing aides and care aides, could not be achieved.

## 3 THE STUDY

### 3.1 Aim

We carried out a qualitative study to assess burdens placed on and consequences of the COVID‐19 pandemic on nursing home staff.

### 3.2 Design

This study is a qualitative descriptive study. We decided to use a qualitative approach for two reasons. First, qualitative approaches, allow an insight view and a more ‘thick description’ than quantitative approaches (Holloway & Galvin, 2017). And second, this design was chosen to compare different professional perspectives by closely examining the data (Kim et al., 2017).

### 3.3 Sample/participants

The participants were recruited from five nursing homes in two Austrian provinces (Styria and Carinthia). Nursing home directors in the corresponding nursing homes acted as gatekeepers for participant recruitment. To obtain a representative sample, a purposeful strategy was chosen based on a sampling plan (Hussy et al., 2010). The sampling plan was developed on the basis of the national distribution of nursing staff in nursing homes (Statistik Austria, 2018) according to the staff qualifications (nurse, nursing aid, care aid), sex (female, male), age (<40, 40–54, ≥55 years) and the location of the nursing home (either rural or urban), which was defined according to Austrian law.

Inclusion criteria for the participants were that they: (1) were capable of consent, (2) could understand and speak German, (3) were older than 18 years, (4) worked as a nurse (N), nurse aid (NA) or care aid (CA) and (5) worked on the frontline directly with the residents during the COVID‐19 pandemic. The written informed consent was obtained from all participants, as well as their nursing directors. We followed the Consolidated criteria for Reporting Qualitative research (COREQ).

### 3.4 Data collection

The first and last author conducted the individual interviews between June and September 2020. Both authors are nursing scientists with previous experience working in a nursing home. The last author, an experienced interviewer, trained the first author and performed training interviews with her.

Between mid of March and 04 May 2020, all nursing homes were closed, due to the national visit ban. After 04 May 2020 relatives were allowed to access the nursing homes again, with specific interventions with regard to infection prevention and control (Federal Ministry for Social Affairs, Health, Care and Consumer Protection, 2020a): they had to make an appointment, one visit per resident, continuously wearing a masks, keeping distance and access to the resident's room was forbidden. Therefore, visits were either allowed outside if possible, or in specific visitor zones. After the 9. June visits were allowed again in the resident's room, without wearing a mask, etc (Federal Ministry for Social Affairs, Health, Care and Consumer Protection, 2020b).

The participants were informed of the interviewer's educational and professional background as well as the purpose of the interview. No working or personal relationship existed between the participants and the interviewers.

To assess the demographics (e.g. age, sex), professional qualification, working experience, experience with highly infectious disease and professional contact with suspected or COVID‐19‐affected residents, a standardized questionnaire was completed by the participants before the interviews started.

Interviews were held using a semi‐structured interview guide based on a questionnaire that had been previously used to assess burdens placed on nursing staff (Koehler & Meyer, 2017). The interview guide consisted of introductory, transition, key, final and summary questions. Key questions were asked to assess the quantitative (e.g. too much work, too little time) and qualitative burdens (any burden not related to workload or time restrictions) on the nursing staff, the work organization (e.g. availability of personal protective equipment [PPE]) as well as the social working environment (e.g. team atmosphere) during the pandemic (Koehler & Meyer, 2017). More specifically, if participants reported additional tasks, but did not perceived them as too much work or too less time to perform them, these reported additional tasks were assigned to the qualitative workload. We also asked the participant to describe the physical, psychological and social consequences of the COVID‐19 pandemic in questions that were also based on those included in the above‐mentioned survey (Koehler & Meyer, 2017). We closed the interviews by summarizing the main statements and asking the participant if they had anything further to add.

The interview guide was tested with the first interview, and no adaptions had to be made. The interviews took place at the nursing home in which the participants worked and in a separate and quiet room. Each interview was audio‐recorded, transcribed verbatim, and the data were stored on a password‐protected university server. A professional company transcribed all interviews semantically based on transcription rules (Dresing & Pehl, 2018) with the exception of three interviews, which were transcribed by us. The interviews took between 20 min and 1 h and 10 min.

### 3.5 Ethical considerations

This study was conducted in accordance with the Declaration of Helsinki and the ’Good Scientific Practice’ guidelines of the respective university, which also ethically approved the study.

### 3.6 Data analysis

We used a qualitative content analysis method to perform data analysis (Mayring, 2010; Schreier, 2012) with a combined inductive and deductive coding frame. This method is specifically useful for analysing data providing answers to descriptive research questions and to categorize and reduce these data (Mayring, 2010; Schreier, 2012). We followed the eight steps defined by Schreier (Schreier, 2012).

The coding frame consisted of deductively defined main categories (quantitative/qualitative burden, work organization, social working environment and physical, psychological, social consequences), and inductively generated first level and second level subcategories. Subcategories were generated by paraphrasing each text passage that was assigned to a main category, streamlining the paraphrased text, comparing these paraphrased texts to identify similar content and streamlining the paraphrased texts repeatedly, if necessary. Each subcategory was provided with a definition, anchor examples and coding rules.

The first and third author developed the coding frame based on the first three interviews. To test the coding frame and search for agreement, thematic segmentation was performed, and the coding frame was blind‐tested with the second author based on two interviews. After all three authors performed and agreed upon the final coding frame, it was applied to the entire dataset (main analysis). One‐third of the dataset was categorized by two authors independently, with an intercoder reliability of about 90%. Discrepancies were discussed until an agreement was reached. Finally, the text segments in the subcategories were summarized. To compare and contrast the perspectives of different nursing home staff, descriptive group comparisons were performed quantitatively and qualitatively (Schreier, 2012). The data analysis was supported by the software Maxqda2020. To quantitatively present the qualitative data, the frequencies of participants with their respective codes were calculated and compared between the subgroups of care aides, nursing aides and nurses.

### 3.7 Rigour

To establish credibility, the respective interviewer summarized the main messages at the end of each interview. This provided an opportunity for clarification and further comments by the interviewee. Member checking was offered by sending the transcripts via an encrypted webpage to the participants (Goldblatt et al., 2011). This offer was accepted by about half of the interviewees, but no changes were requested.

The coding frame draft was pilot tested in two interviews by blind coding to ensure its validity and confirmability (Schreier, 2012). The clear definitions of the subcategories with anchor rules and signal words led to the achievement of high levels of intercoder reliability. The coding framework could be accurately applied to the remaining interviews, and codes were relatively evenly distributed among the subcategories, indicating that data saturation had been reached (Schreier, 2012). To facilitate the transferability of the results (Korstjens & Moser, 2018), the similarities and differences among the respective subcategories for different professional groups were investigated.

### 3.8 Findings

Our sampling plan led us to select eight nurses, eight nursing aides and two care aides, distributed by geographical location, sex and age (Table 1).

**TABLE 1:** Sample characteristics

	Nursing home staff (*N* = 18)
Carinthia % (*n*)	50 (9)
Urban region % (*n*)	50 (9)
Female % (*n*)	77·8 (14)
Mean age in years (SD)	41 (9·8)
Qualification % (*n*)	
Nurses	45·4 (8)
Nursing aides	44·4 (8)
Care aides	11·1 (2)
Work experience % (*n*)	
<5 years	22·2 (4)
5–10 years	33·3 (6)
11–20 years	27·8 (5)
>20 years	16·7 (3)

The majority (61.1%, *n* = 11) of the participants stated that they never/rarely had contact with infectious diseases during their work before the COVID‐19 pandemic. Most participants reported never/rarely having had contact with COVID‐19‐suspected or ‐affected cases (Table 2).

**TABLE 2:** Contact per month with COVID‐19‐suspected or ‐affected residents

	Nursing home staff (*N* = 18)				
	March	April	May	June	July
Never/rarely	77·8 (14)	77·8 (14)	88·9 (16)	83·3 (15)	88·9 (16)
Sometimes	11·1 (2)	0	0	11·1 (2)	11·1 (2)
Often/very often	11·1 (2)	22·2 (4)	11·1 (2)	5·6 (1)	0

Burdens on and consequences for nursing staff—results of the qualitative analysis.

Table 3 gives insights into the categories and subcategories with examples of the respondent's statements.

**TABLE 3:** Categories and subcategories with examples of respondents statements

Category	Quote
*Quantitative work load*	
Additional time resources needed with regard to PPE	“If you were in a hurry or had an “emergency”, then you had to go to a resident. And then you had to completely alter your routine. Especially during the night shift, you went from room to room. And every time you had to change the PPE, you realized that, well, a tour does not take 1.5 h, it takes 2.5 h, right?”(N8)
More time to care for individual residents	“And then you have time again. Yes, for the residents, we also had partly a few minutes to chat with them. Which was pleasant” (N8)
Less time	“We have two hours with activity coordinators every day as well. They do a lot there. … And you also notice that some people like to be kept busy, but there really is not enough time. And you noticed that at the time when there was no one, when there was NO ONE there. …I think there is also a lack of staff, so that you simply have someone who really sits down or plays Ludo” (CA1)
*Qualitative work load*	
Pandemic as an exceptional situation	“It was what I do not think we all expected. It was something new. We had no experience. Otherwise, we are prepared for a lot, but we were not prepared for that. There were new changes every day, and there were new orders that we had to follow every day. And yes, there was a lot of new stuff” (N3)
Residents not burdened	“… the residents were understanding. They knew some things. They listened to the news with me or with us and read to us from the newspaper, or told us what's going on. They knew … that it was a dangerous situation” (N5)
Burden on the residents with regard to the mandatory interventions	“In general, for those already suffering from dementia, the decline went even faster. Above all, we had a lady who lost herself. She was a completely family person, and the family was there all the time and every day. And then that was all over. And from that day on, she simply detached herself and was lost” (NA7)
Residents understood and accepted the interventions	“So I have to be honest with you, and I really have to salute the residents. They really went through everything …. They really accepted it the way it was. I did not experience it any other way. So I would have expected questions like what are you doing there, why do you look like that, or have we landed on Mars now? … But nothing like that. Really, they just accepted it. That's the way it is” (CA2)
Dealing with relatives	“A relative came to see her mother. Unannounced. The relatives knew about the handover times in our nursing home. And then, during this handover time, the visit took place. The resident was found outside with relatives. There wasn't just one person there. There were two people. One kept the distance, but not the other one. They were standing at the end of one corridor. Yes. Both were asked if they knew that the visit would endanger the safety of all residents and staff. Yes. Of course, we asked them if they also understood the importance of complying with the visiting restrictions. The two relatives showed no understanding….” (N6)
Additional tasks required due to the pandemic to care for the residents	“… people want more to drink. This was not because they were feeling thirsty, because there were drinks on the table. Normally, they help themselves. But then they said, I have nothing to drink to anymore. And if you tell them that there are drinks on the tables, they answer that the jug is too full and that it's too difficult to pour. They changed unconsciously very much during that time” (NA1)
Use of new technologies	“Yes, video calls cannot be one hour, because we have 80, 86 residents in the house. … Then there are relatives at home, many sons and daughters are already over 60 and cannot do that either. Then we had to make sure that a grandchild could help at them home” (NA5)
*Work organization*	
Information from the multiprofessional team	“A video was made and sent that out by the hygienist. The staff were able to repeatedly have a look at this video on their ward, (…), how do I put this on correctly” (N3)
Supported by their organization	“And then we received the feedback: Thank you for the work that you do. We need to keep sticking together” (NA7)
“Helping hands” from outside	“So it's really that all work together. The entire health care system has worked together. …And other caregivers, like the ones from the day care center that was closed, had the chance to show what long‐term care is actually like (…). So it was funny. Yes. And then, little by little, conversations started to take place and we learned a lot more about each other” (CA2)
More scope for action	“So there was scope of action and the doctors also gave us that scope of action. Because the doctors also said they would only come in an emergency. Accordingly, we were allowed to make some of the decisions ourselves. Then, from a distance, the doctors said it was all right” (N8)
Little scope of action	“So we just had a certain wheel. Leave for vacation days or days off that had already been planned in the past were not given during this time. But there needed to be as little contact as possible” (NA6)
Decision making	“If you send someone to the hospital, just because the resident falls down, they have to be in quarantine for 14 days. And then you think about it five times. And, therefore, you need experience. You have to stand up and say, (…), I have now done this and that. There is no danger right now in delaying, and the resident should stay here for now. And then you have to be able to deal with this decision, because it means that the resident stays at the institution” (N1)
*Social working environment*	
Cooperation and communication with the supervisors	“In the meantime, the nursing home's manager came to the handover and praised us for our performance” (NA3)
*Physcial consequences*	
Physical consequences mainly due to wearing masks	“It's just with the masks. They put a lot of strain on you, and I can speak for myself. I really did a lot of sports before the coronavirus pandemic. Always in my whole life. Right now, I do not. And I could not either. If I work eleven hours, I'm dead in the evening. As I said, we all have headaches regularly.(…) in the whole team. It is like that. And you have coughing fits. Weird coughing fits from the masks. It's really weird. And we just think that comes from the masks, because of experiences” (NA2)
Tiredness and exhaustion	“You go home, you take a shower, you sleep, you go back to work. And the fifth day is still okay, and from the sixth day on you just function, I think” (N6)
*Psychological consequences*	
Positive effects on their psyche	“(…), family became again more consciously, also for me. Because I never saw my parents for these two months. Then you know again what you have. And how important the whole thing (family) is. (…) One thinks much more about the whole. What do I have and what do I really need in life?” (N2)
Uncertainty because of the situation	“So in the beginning, there was great uncertainty. A huge uncertainty. Because simply no one knew what was going to happen now. (…)No one has known, how and what? How do we go on? What do we do? I do not know, do I have to go into quarantine or not? Or can I still go to work or am I allowed to go to work or why do I have to go to work? These were also questions. Yes, it was difficult” (N2)
Stress	“And you notice that because of all the pressure and stress. You pass it over. It's difficult” (NA2)
Mentally exhausted	“I was at home and really started to cry. Because my nerves just felt like they could not take anymore. Hopefully it's over soon, because otherwise I'll break, I thought, from the whole thing” (N5)
Fear of getting infected	“And that fear of getting infected, yeah, you still have fear. It's always there” (NA3)
Fear for the residents	“But there is just the fear. Because I do not want to infect anyone in any way that will cause them to die because of it. So that was my fear” (CA2)
*Social consequences*	
Social distance and reduction of contacts	“Because you do not want to bring the infection into the nursing home. Therefore, we all scaled back our social contacts radically” (NA2)
More intensive contact	“And so I have to say, we privately as a family enjoyed the lockdown more. The time has allowed us to be much more aware of things. And yes, we have done much more at home. Because you were not allowed to go out, you just did a lot more with the children, with the family, right” (NA7)
Family environment as a source of support	“They cooked, they shopped. And every day at three in the afternoon they rang the bell to tell my son that his ice cream was outside the door” (NA6)

#### 3.8.1 Quantitative work load

The nurses had to provide *additional time resources with regard to PPE*. As an example they had to ensure that the PPE were correctly handled. Moreover, they had to check for compliance with the additional hygiene measures.

Due to the fact that fewer appointments were made for various reasons, including the cancellation of therapies, all nursing caregiver groups said that they were able to devote *more time to care for individual residents*, which was perceived as very pleasant.

And then you have time again. Yes, for the residents, we also had partly a few minutes to chat with them. Which was pleasant (N8).

The participants perceived working during the lockdown as calmer and more relaxed. They repeatedly emphasized the fact that this calm atmosphere was due to the lack of visitors. These additional time resources also became available because the wards were better staffed than usual, in part because vacation time or compensatory hours could not be taken.

However, since certain professional groups such as activity coordinators were not available during lockdown, nursing staff had to take over certain activities from these occupational groups, such as amusing and engaging the residents. This resulted in nursing aides and especially care aides having *less time* for daily tasks.

#### 3.8.2 Qualitative work load

All groups of participants stressed that *information from outside* sources such as the media placed an additional burden on them. They mentioned that the residents almost panicked when the nursing home staff started to wear masks, because they feared that bad things were happening due to the information they had received from the media and believed that the coronavirus had now arrived in Austria. The nurses stated that they had not expected this and needed to take time to explain why they were wearing a mask, namely, because wearing a mask is also mandatory when a viral infection (e.g. norovirus) is present. For that reason, they did not think to inform the residents in a timely manner.

One major topic was the *burden on the residents with regard to the mandatory interventions*, such as PPE and isolation.

Our residents with dementia often woke up screaming at night because they saw us with the mask on (N2).

The participants also described (1) feeling imprisoned, (2) the effects of the visiting ban, (3) the effects of wearing PPE and (4) conflicts that arose between the residents due to boredom. With regard to feeling imprisoned, the participants said that the residents wanted to go out and make their common rounds and that, the longer it took, the more restless the residents became. The participating nursing home staff also described that the status of the residents declined due to the visiting ban.

*Dealing with relatives* was one main burdensome aspect that the participating nursing home staff, and especially the nurses and nursing aides, experienced. They described how relatives ignored the obligation to wear a mask or tried to get into the nursing home through the door of the terrace. Others described that, at the start of the pandemic, relatives even threatened to report them to the newspaper or the police if the relatives do not get access to the nursing home.

Another burdensome aspect that the nursing home staff reported were the *additional tasks required due to the pandemic to care for the residents*. Additional tasks with regard to the residents were to explain and remind the residents of the implemented measures, to engage and amuse the residents, to talk to the residents, to calm them down and to fulfil the residents' need for more attention during the pandemic. Other additional tasks that were mentioned were visitor management, the organization/monitoring of social distancing/isolation, the monitoring of symptoms (e.g. fever measurement), desinfection, more documentation and the organization of the meals/laundry.

#### 3.8.3 Work organization

*Information received from the nursing home management* was applied in practice in different ways: (1) verbal instructions from the nursing teams, (2) information made available on an information stand (Infopoint) in the facility, (3) information sent by e‐mail regularly once a week with information for the next week, (4) information sent via the electronic information system with daily updates and (5) written information available in guidelines which were handed out on the wards (receipt confirmed by signature). The information was generated by nursing care managers and nursing home managers and mainly concerned protective equipment, hygiene rules and structural changes (e.g. in the daily routine). Nurses and nursing aides generally felt that they had been clearly informed by their organization.

The nurses said that they felt well *supported by their organization*. In the nursing homes, the necessary resources (e.g. disinfectants) were available in sufficient amounts, which was perceived as very positive by the nursing staff. In one facility, the staff were even allowed to take disinfectants home for their private use. In addition, the facilities assigned persons who could be reached at any time or who could be contacted if questions arose (e.g. regarding hygiene). The nursing staff made particularly positive statements about the appreciation they had received from the organization.

And then we received the feedback: Thank you for the work that you do. We need to keep sticking together (NA7).

Moreover, they mentioned receiving positive feedback from the nursing care manager at the handover, receiving encouraging e‐mails, receiving a snack or being paid a bonus.

#### 3.8.4 Social working environment

With regard to the *cooperation and communication within the team*, all of the participants stated that a very good working atmosphere existed during the pandemic. Participants repeatedly emphasized the fact that ‘in times of crisis, people stick together’ (NA5). In the nursing homes, teams were formed to minimize the interacting time between colleagues. Working in these teams (two teams were established in most nursing homes) with the same people was felt to strengthen cohesion. The participants said that this gave them more opportunities to talk to each other and that mutual motivation and support were prioritized. This positive working atmosphere was promoted by better staffing during the lockdown.

Focussing on the *cooperation and communication with the supervisors’* discussions often dealt with compliance with hygiene measures and the importance of not having any COVID‐19 infections in the nursing home. One nurse said that the supervisors were always concerned when it came to serious issues. When it came to minor issues, they were rather annoyed or seemed stressed. Nursing aides felt it was positive to receive praise from the nursing home management.

#### 3.8.5 Physical consequences

Some participants stated that they *did not experience any physical consequences* as a result of working as a member of nursing staff during the COVID‐19 pandemic. Others stated that they experienced *physical consequences mainly due to wearing masks*, such as skin redness, acne and rashes, feeling overheated, breathing in stale air, soreness on pressure points, headaches and suffering from coughing fits, and that wearing the mask was also perceived as very strenuous. Two nursing aides also reported having redness and irritation on the hands due to the constant disinfection. Nine participants also described *tiredness and exhaustion as physical consequences*.

You go home, you take a shower, you sleep, you go back to work. And the fifth day is still okay, and from the sixth day on you just function, I think (N6).

#### 3.8.6 Psychological consequences

The nursing home staff mentioned that the feeling of *uncertainty because of the situation* was one main psychological concern. They cited several reasons for this uncertainty, including the lack of information about the disease itself, unclear instructions on how to put on or take off PPE, the need for further procedures and interventions in the nursing homes and the lack of testing possibilities.

One more psychological consequence was the *feelings of fear*. Nurses mentioned experiencing fear at the beginning of the pandemic *in general*. Nurses and nursing aides also stated that they were *afraid for their own family* because of the risk of infection, whereas the care and nursing aides were afraid of *becoming infected themselves*. The majority of the participants independent of their level of professional qualification said that they experienced *fear of infecting the residents*.

But there is just the fear. Because I don't want to infect anyone in any way that will cause them to die because of it. So that was my fear (CA).

#### 3.8.7 Social consequences

Overall, one care and one nursing aid and two nurses stated that they *did not experience social consequences* due to the pandemic.

The most frequent mentioned social impact on the nurses, nursing and care aides was the *social distance and reduction of contacts* in general. This social distance was mostly mentioned in combination with other factors. Both the social distance and reduction in the number of contacts were exercised out of fear of infecting the family and also to protect the residents from an infection. Two nurses named having *more intensive contact* primarily with family during the lockdown and mentioned having contact with their parents most frequently. In the case of three of the nurses interviewed, the partner or *family environment* was most frequently mentioned *as a source of support*.

They cooked, they shopped. And every day at three in the afternoon they rang the bell to tell my son that his ice cream was outside the door (NA6).

Burdens on and consequences for nursing staff—results of the quantitative analysis.

Care aides reported more often that they had more time for the residents (Table 4). This result contrasts with that for the nursing aides, whereby the majority stated that they had less time in general. The time constraints associated with putting on/taking off PPE were only described by care aides and nurses.

**TABLE 4:** Categories and subcategories of work load, organization and environment distributed by professional qualification

	Care aides (*n* = 2)	Nursing aides (*n* = 8)	Nurses (*n* = 8)
Quantitative work load % (*n*)			
More time for the residents	100·0 (2)	50·0 (4)	75·0 (6)
Time aspects of PPE	50·0 (1)	0	50·0 (4)
Less time	50·0 (1)	62·5 (5)	37·5 (3)
Qualitative work load % (*n*)			
COVID as exceptional situation	100·0 (2)	25·0 (2)	87·5 (7)
Information from outside	50·0 (1)	37·5 (3)	25·0 (2)
Situation for residents			
Not/less burdensome	50·0 (1)	25·0 (2)	25·0 (2)
Burdensome	50·0 (1)	37·5 (3)	37·5 (3)
Interventions for residents			
Not/less burdensome	50·0 (1)	62·5 (5)	50·0 (4)
Burdensome	100·0 (2)	87·5 (7)	87·5 (7)
Dealing with relatives			
Relatives as a challenge	50·0 (1)	37·5 (3)	100·0 (8)
Relatives as a resource	50·0 (1)	37·5 (3)	25·0 (2)
Understanding the relatives	100·0 (2)	12·5 (1)	12·5 (1)
Additional tasks due to COVID‐19			
Additional tasks for resident's care	100·0 (2)	100·0 (8)	100·0 (8)
Use of new technologies	100·0 (2)	37·5 (3)	50·0 (4)
Contact with external experts	0	0	50·0 (4)
Organization of PPE	0	12·5 (1)	25·0 (2)
Work organization % (*n*)			
Information			
from the management	0	50·0 (4)	75·0 (6)
from the team	50·0 (1)	0	25·0 (2)
Support by the organization	100·0 (2)	62·5 (5)	87·5 (7)
Helping hands from outside	50·0 (1)	0	25·0 (2)
Scope for action			
More	50·0 (1)	37·5 (3)	62·5 (5)
Less	50·0 (1)	50·0 (4)	25·0 (2)
Decision‐making behaviour	0	0	37·5 (3)
Social working environment % (*n*)			
Within the team	100·0 (2)	100·0 (8)	100·0 (8)
With the supervisors	50·0 (1)	50·0 (4)	37·5 (3)

Care aides and nearly all nurses also described the COVID‐19 pandemic as an exceptional situation for their daily nursing practice. Nearly all qualification groups mentioned that the interventions placed heavy burdens on the residents. All nurses reported that dealing with relatives presented them with challenges. All of the interviewed nursing staff members stated that they needed to perform additional tasks due to the COVID‐19 pandemic to adequately care for the residents. Only nurses mentioned that maintaining contact with external experts, such as doctors, represented an additional task which increased their work load. Table 5 displays the categories and subcategories of consequences distributed by professional qualification.

**TABLE 5:** Categories and subcategories of consequences distributed by professional qualification

	Care aides (*n* = 2)	Nursing aides (*n* = 8)	Nurses (*n* = 8)
Physical consequences % (*n*)			
No physical consequences	50·0 (1)	25·0 (2)	25·0 (2)
Tiredness and exhaustion	50·0 (1)	37·5 (3)	62·5 (5)
Due to PPE	100·0 (2)	75·0 (6)	50·0 (4)
Psychological consequences % (*n*)			
No psychological consequences	50·0 (1)	25·0 (2)	37·5 (3)
Positive effects on their psyche	50·0 (1)	25·0 (2)	37·5 (3)
Uncertainty			
because of the situation	100·0 (2)	87·5 (7)	62·5 (5)
regarding the future	50·0 (1)	12·5 (1)	25·0 (2)
Stress	100·0 (2)	37·5 (3)	62·5 (5)
Psychological exhaustion	0	25·0 (2)	62·5 (5)
Missing freedom	100·0 (2)	25·0 (2)	25·0 (2)
Fear			
in general	0	0	25·0 (2)
for my family	0	25·0 (2)	50·0 (4)
to infect myself	50·0 (1)	50·0 (4)	0
for the residents	100·0 (2)	50·0 (4)	37·5 (3)
Social consequences % (*n*)			
No social consequences	50·0 (1)	12·5 (1)	25·0 (2)
Support by the family	50·0 (1)	12·5 (1)	37·5 (3)
More intensive contact	50·0 (1)	12·5 (1)	25·0 (2)
Social distance and reduction of contacts	100·0 (2)	100·0 (8)	100·0 (8)

## 4 DISCUSSION

The objective of this study is to assess burdens placed on and consequences of the COVID‐19 pandemic on nursing home staff. Our results show that the qualitative work load and work organization were major concerns among nursing staff during this pandemic. Regarding the qualitative work load, the main described aspects were the additional tasks that needed to be performed due to the pandemic to care for the residents, the fact that the residents were placed under stressed due to the interventions and that dealing with the relatives presented a significant challenge. Nursing home staff reported psychological consequences, such as uncertainty, fear and stress, as major effects of the COVID‐19 situation.

Regarding the quantitative work load, nursing aides and care aides interviewed in our study reported that they had less time to perform daily tasks, whereas the nurses mentioned challenges associated with correctly handling as well as (training in) putting on and taking off PPE. These findings are similar to those reported by another study, which reported that nurses were concerned about wasting their time with these tasks, such as wearing PPE (Galehdar et al., 2020). On the other hand, members of all professional groups said that they had more time for the residents because there were fewer appointments due to, for example cancellation of therapies. This is an interesting finding, because another study pointed out that the nurses were concerned about the fact that the number of caregivers as compared to patients was relatively low (Sun et al., 2020). However, the latter study was conducted in a hospital, and only nurses who provided care for COVID‐19 patients were interviewed (Sun et al., 2020). Furthermore, our study was performed after the first lockdown, when it was still unclear whether a vacation ban would go into effect. That means that more staff were available. This might explain the differences in the results.

In our study, the qualitative work load was a major concern for nursing staff during this pandemic. The study participants particularly expressed concern about the additional tasks that arose due to the pandemic to care for the residents, to engage and amuse them, and to calm them down. Our results are similar to the findings of another study, which highlighted the need to treat the patient and not just the disease by providing patients with emotional support (Liu et al., 2020).

The protective measures that became necessary due to the pandemic were perceived differently by the residents. Many residents seemed to understand the need for these measures, and the carers indicated that residents did not feel restricted. Other residents, and especially residents with cognitive impairments, found the measures with respect to PPE very stressful. Their level of understanding for the measures also decreased as the period of restrictions lengthened. Although it is essential to wear PPE during such a pandemic to protect both residents and staff, this result underlines the heterogeneity of the nursing home population and shows that individual protective care concepts are necessary in exceptional situations. Regularly explaining the reasons for the measures to the residents and offering alternatives that enable social participation would also be important in these situations. One strategy that can provide residents with some orientation is to wear special laminated badges that include the name and the face on, for example the gown. This was already done by several healthcare workers allaround the world (Johnson, 2020).

Nurses reported that the visiting ban caused the status of residents to decline. Other studies show that residents experienced loneliness and emotional burdens as consequences of the COVID‐19 visiting restrictions (Liu et al., 2020; Sarabia‐Cobo et al., 2021). White et al. (2021) even reported an increase in the numbers of deaths among otherwise stable residents after the beginning of the visitation/activity restrictions (White et al., 2021). These findings are interesting, because we know that up to 42% of residents, regardless of COVID‐19, feel lonely (Victor, 2012). However, the pandemic had an unexpected impact on all persons involved (Gould & Hantke, 2020). This might explain the focus placed on physical aspects and neglect for the psychological and social components and consequences of this pandemic. To prepare for future pandemics, there is a desperate need to establish guidelines that include recommendations that help to enhance the residents' psychological and social wellbeing in addition to their physical wellbeing.

Dealing with relatives was also mentioned as one main aspect in our study that was experienced as burdensome, specifically by the nurses and nursing aides. Relatives are assigned key persons in nursing homes, because they are in a unique position to understand, articulate and support the emotional, social and health‐related needs of the residents (Reinhard et al., 2008). As a result of the visiting restrictions, relatives could no longer visit and support the residents; instead, the caregivers had to inform them of these restrictions. Relatives tended to reject these restrictions, which meant that conflicts and discussions arose with the caregivers. In one nursing home, the management took over the task of providing information and instructions for the relatives, which the caregivers in this facility perceived as a great relief. This approach could also be recommended in other nursing homes to relieve nurses on the frontline as much as possible.

Fear was mentioned as a negative psychological effect of the COVID‐19 pandemic. The participants reported that they were afraid of infecting themselves or their families. This is also reported by other studies carried out in hospital as well nursing home settings (Galehdar et al., 2020; Liu et al., 2020; Sarabia‐Cobo et al., 2021; Sun et al., 2020; White et al., 2021). However, one of the biggest fears expressed was the fear of infecting the residents; this fear was expressed by nearly all of the participants, independent of the professional qualification.

### 4.1 Limitations

We note some limitations of this study. First, the full purposive sampling plan could not be achieved, as we lacked one female nurse 55 years or older, one female nursing aid from urban area younger than 40 years and one male care aid 40–54 years old in our sample. Instead, we interviewed three additional nurses. However, this study findings provide representative insights into the burdens experienced by a heterogeneous nursing home staff population. Second, the interviews were conducted retrospectively after the first COVID‐19 wave. This might have influenced the experiences and perspectives of the interviewed staff members in terms of recall bias. However, as this study allowed us to collect data through individual face‐to‐face interviews, these data also show strong statistical and scientific rigour. We also ensured the validity of the data by applying methodological triangulation. One more limitation is that we did not collect data on the distribution of the nursing staff in the five nursing homes. However, we based our sampling plan on national statistical information, assuming to get a representative insight to the situation of the nursing home staff during the pandemic. In addition, as we included nursing home staff with different professions, we were able to show a holistic picture of the processes taking place in the nursing home during COVID‐19 pandemic.

## 5 CONCLUSION

This was the first study to examine the burdens placed on different professional nursing home groups and the consequences as a result of the COVID‐19 pandemic in depth. We could show that the qualitative workloads were assessed and perceived differently by staff members. Most nursing home care staff members experienced the accompanying interventions as both physically and psychologically challenging. We highly recommend that support be provided for nursing home staff during such pandemics by offering, for example specified PPE breaks. To enhance the wellbeing of both staff and residents, as well as to decrease psychological stress and fear during such challenging times, individually tailored programs need to be established. Although this study had some limitations, a high level of scientific rigour could be achieved. We recommend that further research be conducted on an ongoing basis, rather than retrospectively.

## CONFLICT OF INTEREST

The authors declare no conflict of interest.

### PEER REVIEW

The peer review history for this article is available at https://publons.com/publon/10.1111/jan.15193.

## Data Availability

Due to legal issues, data can not be made available.

## References

[jan15193-bib-0001] Benzinger, P. , Kuru, S. , Keilhauer, A. , Hoch, J. , Prestel, P. , Bauer, J. M. , & Wahl, H. W. (2021). Psychosocial effects of the pandemic on staff and residents of nursing homes as well as their relatives‐a systematic review [Psychosocial Auswirkungen der Pandemie auf Pflegekräfte und Bewohner von Pflegeheimen sowie deren Angehörige – Ein systematisches review]. Zeitschrift für Gerontologie und Geriatrie, 54(2), 141–145. 10.1007/s00391-021-01859-x 33624143 PMC7901511

[jan15193-bib-0002] Dresing, T. , & Pehl, T. (2018 & , Praxisbuch: Interviews transcription & analysis‐guidance and rule system for qualitative researchers (8th ed.). Author.

[jan15193-bib-0003] Federal Ministry for Social Affairs, Health, Care and Consumer Protection . (2020a). Empfehlungen zur schrittweisen Lockerung der aufgrund der COVID‐19 Pandemie erlassenen Besuchsbeschränkungen in Alten‐ und Pflegeheimen ab 4. Mai 2020. Author.

[jan15193-bib-0004] Federal Ministry for Social Affairs, Health, Care and Consumer Protection . (2020b). Empfehlungen zur schrittweisen Rückkehr zum Alltag in Alten‐ und Pflegeheimen und teilstationären. Einrichtungen ab 9. Juni 2020. Author.

[jan15193-bib-0005] Galehdar, N. , Kamran, A. , Toulabi, T. , & Heydari, H. (2020). Exploring nurses' experiences of psychological distress during care of patients with COVID‐19: A qualitative study. BMC Psychiatry, 20(1), 489. 10.1186/s12888-020-02898-1 33023535 PMC7538040

[jan15193-bib-0006] Goldblatt, H. , Karnieli‐Miller, O. , & Neumann, M. (2011). Sharing qualitative research findings with participants: Study experiences of methodological and ethical dilemmas. Patient Education and Counseling, 82(3), 389–395. 10.1016/j.pec.2010.12.016 21257280

[jan15193-bib-0007] Gould, C. E. , & Hantke, N. C. (2020). Promoting technology and virtual visits to improve older adult mental health in the face of COVID‐19. The American Journal of Geriatric Psychiatry, 28(8), 889–890. 10.1016/j.jagp.2020.05.011 32425468 PMC7227501

[jan15193-bib-0008] Harris‐Kojetin, L. , Sengupta, M. , Park‐Lee, E. , & Valverde, R. (2013). Long‐term care services in the United States: 2013 overview. Vital and Health Statistics, 3(37), 1–107. 26158640

[jan15193-bib-0009] Holloway, I. , & Galvin, K. (2017). Qualitative research in nursing and healhcare (4th ed.). Wiley & Sons.

[jan15193-bib-0010] Hussy, W. , Schreier, M. , & Echterhoff, G. (2010). Qualitative Forschungsmethoden. In Forschungsmethoden in Psychologie und Sozialwissenschaften für Bachelor. Springer.

[jan15193-bib-0011] Johnson, A. (2020). Health care worker covered in protective gear laminates smile on chest to comfort patients. National Broadcasting Company.

[jan15193-bib-0012] Kabir, Z. N. , Boström, A.‐M. , & Konradsen, H. (2020). In conversation with a frontline worker in a care home in Sweden during the COVID‐19 pandemic. Journal of Cross‐Cultural Gerontology, 35(4), 493–500. 10.1007/s10823-020-09415-7 33015728 PMC7533166

[jan15193-bib-0013] Kim, H. , Sefcik, J. S. , & Bradway, C. (2017). Characteristics of qualitative descriptive studies: A systematic review. Research in Nursing & Health, 40(1), 23–42. 10.1002/nur.21768 27686751 PMC5225027

[jan15193-bib-0014] Koehler, S. , & Meyer, A. (2017). Psychische Belastung und Beanspruchung. Berufsgenossenschaft für Gesundheitsdienst und Wohlfahrtspflege (BGW).

[jan15193-bib-0015] Korstjens, I. , & Moser, A. (2018). Series: Practical guidance to qualitative research. Part 4: Trustworthiness and publishing. The European Journal of General Practice, 24(1), 120–124. 10.1080/13814788.2017.1375092 29202616 PMC8816392

[jan15193-bib-0016] Liu, Q. , Luo, D. , Haase, J. E. , Guo, Q. , Wang, X. Q. , Liu, S. , Xia, L. , Liu, Z. , Yang, J. , & Yang, B. X. (2020). The experiences of health‐care providers during the COVID‐19 crisis in China: A qualitative study. The Lancet Global Health, 8(6), e790–e798. 10.1016/s2214-109x(20)30204-7 32573443 PMC7190296

[jan15193-bib-0017] Mayring, P. (2010). Qualitative Inhaltsanalyse. VS Verlag für Sozialwissenschaften.

[jan15193-bib-0018] McGilton, K. , Escrig‐Pinol, A. , Gordon, A. , Chu, C. , Zúñiga, F. , Sanchez, M. , Boscart, V. , Meyer, J. , Corazzini, K. , Jacinto, A. , Spilsbury, K. , Backman, A. , Scales, K. , Fagertun, A. , Wu, B. , Edvardsson, D. , Lepore, M. , Leung, A. , Siegel, E. , et al. (2020). Uncovering the devaluation of nursing home staff during COVID‐19: Are we Fuelling the next health care crisis? Journal of the American Medical Directors Association, 21(7), 962–956. 10.1016/j.jamda.2020.06.010 32674829 PMC7287421

[jan15193-bib-0019] Montayre, J. , & Montayre, J. (2017). Nursing work in long‐term care: An integrative review. Journal of Gerontological Nursing, 43(11), 41–49. 10.3928/00989134-20170519-02 28556871

[jan15193-bib-0020] O'Neill, D. , Briggs, R. , Holmerová, I. , Samuelsson, O. , Gordon, A. L. , Martin, F. C. , & The Special Interest Group in Long Term Care of the European Geriatric Medicine Society . (2020). COVID‐19 s the need for universal adoption of standards of medical care for physicians in nursing homes in Europe. European Geriatric Medicine, 11, 1–6. 10.1007/s41999-020-00347-6 PMC7298916 32557250

[jan15193-bib-0021] Reinhard, S. , Given, B. , Petlick, N. , & Bemis, A. (2008). Supporting family caregivers in providing care. In R. G. Hughes (Ed.), Patient safety and quality: An evidence‐based handbook for nurses. Agency for Healthcare Research and Quality (US) https://www.ncbi.nlm.nih.gov/books/NBK2665/ 21328765

[jan15193-bib-0022] Sarabia‐Cobo, C. , Pérez, V. , de Lorena, P. , Hermosilla‐Grijalbo, C. , Sáenz‐Jalón, M. , Fernández‐Rodríguez, A. , & Alconero‐Camarero, A. R. (2021). Experiences of geriatric nurses in nursing home settings across four countries in the face of the COVID‐19 pandemic. Journal of Advanced Nursing, 77(2), 869–878. 10.1111/jan.14626 33150622

[jan15193-bib-0023] Schreier, M. (2012). Qualitative content analysis in practice. Sage Publications Ltd.

[jan15193-bib-0024] Statistik Austria . (2018). Nichtärztliches Gesundheitspersonal 2018 in Krankenanstalten nach Geschlecht. Fachrichtung und Bundesland.

[jan15193-bib-0025] Strang, P. , Bergström, J. , Martinsson, L. , & Lundström, S. (2020). Dying from COVID‐19: Loneliness, end‐of‐life discussions, and support for patients and their families in nursing homes and hospitals. A National Register Study. Journal of Pain and Symptom Management, 60(4), e2–e13. 10.1016/j.jpainsymman.2020.07.020 32721500 PMC7382350

[jan15193-bib-0026] Sun, N. , Wei, L. , Shi, S. , Jiao, D. , Song, R. , Ma, L. , Wang, H. , Wang, C. , Wang, Z. , You, Y. , Liu, S. , & Wang, H. (2020). A qualitative study on the psychological experience of caregivers of COVID‐19 patients. American Journal of Infection Control, 48(6), 592–598. 10.1016/j.ajic.2020.03.018 32334904 PMC7141468

[jan15193-bib-0027] Victor, C. R. (2012). Loneliness in care homes: A neglected area of research? Ageing Health, 8(6), 637–646. 10.2217/ahe.12.65

[jan15193-bib-0028] White, E. , Aiken, L. , Sloane, D. , & McHugh, M. (2020). Nursing home work environment, care quality, registered nurse burnout and job dissatisfaction. Geriatric Nursing, 41(2), 158–164. 10.1016/j.gerinurse.2019.08.007 31488333 PMC7051884

[jan15193-bib-0029] White, E. , Wetle, T. , Reddy, A. , & Baier, R. (2021). Front‐line nursing home staff experiences during the COVID‐19 pandemic. Journal of the American Medical Directors Association, 22(1), 199–203. 10.1016/j.jamda.2020.11.022 33321076 PMC7685055

